# Results of the treatment of bone metastases with modular prosthetic replacement—analysis of 67 patients

**DOI:** 10.1186/s13018-016-0353-6

**Published:** 2016-02-05

**Authors:** Grzegorz Guzik

**Affiliations:** Department of Orthopaedic Oncology, Specialist Hospital in Brzozów, Podkarpacie Oncology Centre, Bielawskiego 18, 36-200 Brzozów, Poland

**Keywords:** Bone metastases, Bone tumor resection, Postresectional bone alloplasty, Modular prostheses, Bone radiotherapy

## Abstract

**Background:**

Surgical treatment of long-bone metastases requires a comprehensive approach. The indications for surgery are based on the patient’s general condition, type and stage of cancer, and survival time expectancy. Tumor modular endoprostheses have been increasingly used. Surgery should provide pain relief and improve the quality of life.

**Methods:**

Between 2010 and 2013, 67 patients with malignant metastases were surgically treated with resection prostheses. We performed a retrospective analysis of the indications for the surgery, its course, the type of the prostheses used, and the implantation techniques applied. We evaluated the most important clinical parameters influencing the postoperative quality of life of the patients.

**Results:**

Breast, prostate, and lung cancers are the most common primary tumors that metastasize to bones. The most common site of the lesions is the proximal femur; sporadically, they do occur in bones distal to the knee and elbow. After the surgery, all the patients could walk, most of them without crutches. The pain, rated on a VAS scale, decreased significantly, and the Karnofsky score improved. We observed that joint mobility and the strength of the muscles in the limbs allowed for normal functioning. Postoperative complications including infections and local tumor recurrences were rarely observed.

**Conclusions:**

The use of modular prostheses is an adequate method of treatment in patients with bone metastases. A radical resection of the tumor, which prevents local recurrences and loosening of implants, gives good outcomes. Reduced joint mobility resulting from muscle attachment cutting is well tolerated and concerns mainly patients that underwent operations on the humerus.

## Background

A significant progress in oncology has resulted in the prolonged survival of patients with myeloma as well as breast, prostate, kidney, and thyroid cancer, but the incidence of bone metastasis has risen. Most patients with bone metastases need a combination of surgical and oncological treatment. The disease is associated with general bad condition, pain, reduced mobility, trouble walking and working, and problems with independent functioning. Often, the patients have to give up their independent living and move to care facilities. In the case of large, lytic tumors posing a risk of pathological fractures and the already existing fractures, radiotherapy is ineffective [1–3].

Bone metastases most commonly affect the axial skeleton involving the vertebral column, ribs, pelvis, and the proximal femur and humerus. In most cases of bone metastases, bone resorption and formation processes coexist. The predominance of one process over the other determines the type of metastasis. Sclerotic lesions, almost exclusively arising from the prostatic carcinoma, are rarely an indication for surgical treatment. Lytic and mixed metastases occur most frequently and pose a risk of pathological fractures when involving load-bearing bones [1, 3, 4].

It is very important to perform the surgery before fracture occurs. Standard radiograms provide sufficient information about the structure of the bone and the risk of a pathological fracture which is quantified in Mirel’s scoring system. In the case of the involvement of the vertebral column or the pelvis, CT scans and MRI prior to the surgery are recommended for the more precise evaluation of the extent of the lesions and thus appropriate planning of the surgery [1, 5].

The qualification for surgery should be multifaceted with account being taken of the age of the patient, general condition and the type, staging, and grading of cancer. Patients in poor general condition and with poor survival prognosis are referred for palliative care. In such cases, it is usually agreed not to perform metastatic tumor resection but to stabilize the fracture, which is followed by radiotherapy. Patients with better prognoses undergo resections of the metastatic tumor, which significantly decrease pain and reduce the risk of local recurrences. The use of tumor endoprosthesis should provide effective and fast pain relief, early mobilization, and longer implants survival. However, the use of megaprostheses has some drawbacks, such as a high risk of infections arising in surgical wounds and reduced functions of the limbs due to damaged muscle attachments [1, 6–8].

## Methods

Over the period 2010–2013 at the Orthopaedic Department in Brzozów, 67 long-bone metastatic tumor resections combined with modular prostheses implantations were performed. The implants used at our department were GMRS, Stryker (28) and MUTARS, Implantcast (39).

We analyzed the medical records of the patients with special attention being given to the type and the stage of cancer, the duration of the disease, the type of treatment, and the prognosis. What was also assessed was the general condition of the patients, the location and the intensity of pain rated by VAS, Karnofsky performance status score in patients, their joint mobility and ability to move, and the provided orthopedic equipment. Before the surgery, radiographic examinations in two projections were conducted. In the cases of particularly large or histopathologically unconfirmed tumors, we carried out CT and MRI scans of the involved regions to assess the size and location of the tumor, bone tissue, and cortical layer condition as well as the involvement of the medullary cavity. No vascular angiography of the tumor was performed. The analysis of preoperative imaging tests always involved precise planning of the surgical approach and the evaluation of the extent of bone and soft tissue resection. Tumor resections were made with a wide margin just like in the cases of primary bone tumors. After the surgery, we assessed the intensity of pain with VAS as well as the limb vasculature and innervation. Rehabilitation records were reviewed considering the time when the walking and postoperative kinesitherapy was started and the provided orthopedic equipment. The passive and active range of joint motion was evaluated 14 days and 3 months after the surgery. We also considered the efficiency of different muscle groups, pain intensity, and the Karnofsky performance status score in patients. On the second, the 14th day, and 3 months after the surgery, radiographic signs were analyzed in the context of the risk associated with recurrence or implant loosening. The patients are under the medical care of orthopedists with follow-up visits repeated at 3-month intervals. The research has been performed in accordance with the declaration of Helsinki. As this retrospective analysis consists of anonymised clinical routine data, the Research Ethics Committee (Okręgowa Izba Lekarska in Crakov, ul Krupnicza ) deems the application for and issue of an Ethics approval not necessary. All the patients gave a written consent to the use of data for research.

## Results

**Fig. 1:**
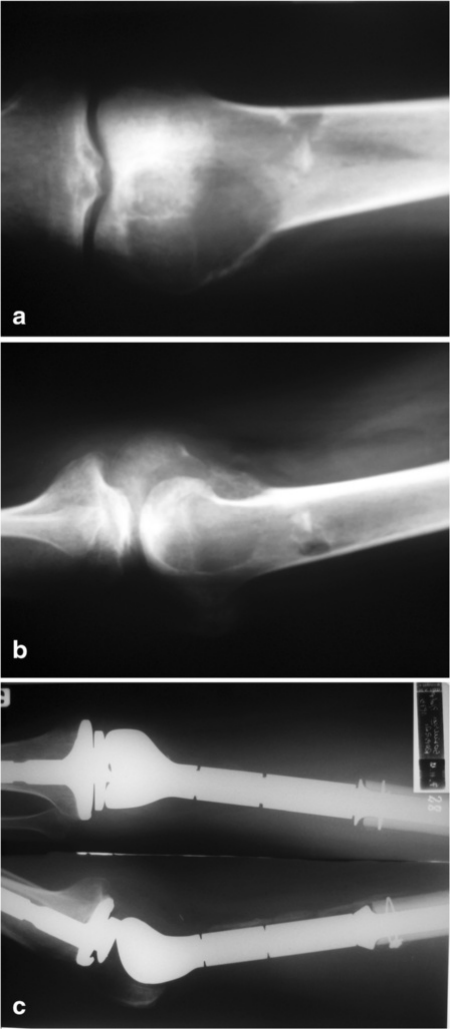
Typical indications for modular endoprosthetic replacement in the distal femur. Bone destruction by metastatic breast carcinoma (**a**, **b**) and radiograph of the modular endoprosthetic replacement (**c**)

Most patients (41) were women, and 26 were men. The average age of women was 67, and of men 69. The mean length of the monitoring period was 2.3 years (range 3.6–1.4 years). So far, 21 patients have died. Pathological fractures were diagnosed in 61 patients. In six patients, the size of the metastasis suggested a high risk of a fracture. In all the patients with no detected fractures, the lesions involved the proximal area of the femur. Lytic lesions were noted in 66 cases (Fig 1), and a sclerotic lesion in one case. Large soft tissue tumors were diagnosed in 52 patients.

**Fig. 2:**
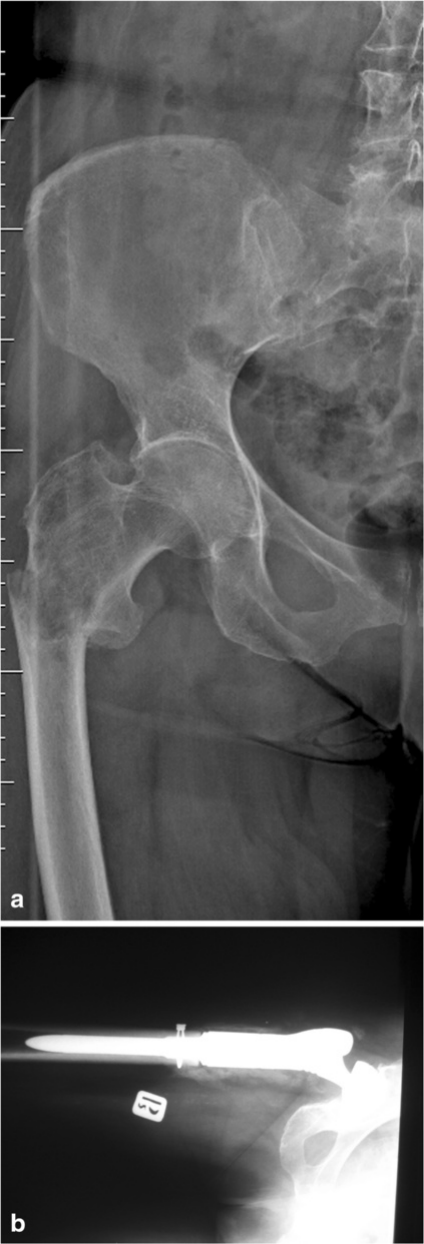
Radiographs of proximal femur destruction by metastatic renal cancer (**a**) and modular endoprosthetic replacement (**b**)

The incidence of primary cancers presented itself as follows: breast cancer (32 patients), myeloma (12 patients), kidney cancer (11 patients), bowel cancer (2 patients), thyroid cancer (2 patients), lung cancer (2 patients), prostate cancer (1 patient), and cancer of unknown primary site (5 cases). The patients in whom the pathological fracture was the first symptom of neoplastic disease underwent biopsy and the surgery was postponed until its results were obtained (Fig 2). In all the five cases, the histopathological examination confirmed the metastatic bone tumor. With regard to the diagnosis, kidney cancer was detected in two cases, and lung cancer in one case. In the remaining two cases, it was not possible to precisely determine the primary site of the neoplasm which revealed itself as adenocarcinoma in the histopathological analysis (Table 1).

**Table 1:** Metastatic cancer type correlated with a modular prosthesis used

Cancer type	Proximal femur	Distal femur	Total femur	Proximal tibia	Proximal humerus	Total humerus
Breast cancer	23	3	1	3	2	
Myeloma	9	1	1	1		
Kidney cancer	6	1			3	1
Colon cancer	2					
Thyroid cancer	2					
Lung cancer					1	1
Prostate cancer	1					
Unknown site	2	1		1	1	

The implanted prostheses included: 2 total humerus prostheses, 7 proximal humerus prostheses, 45 proximal femur prostheses, 6 distal femur prostheses, 2 total femur prostheses, and 5 proximal tibia prostheses (Table 2).

**Table 2:** Type of the prosthesis and the method of bone implantation

	Proximal femur	Distal femur	Total femur	Proximal tibia	Proximal humerus	Total humerus
Implantation with cement	7	6		5	3	
Implantation without cement	38				4	
Trevira mesh implantation	8				5	2
Snap-lock acetabular cup	4		2			
Bipolar acetabular cup	38					
Cemented acetabular cup	7					
Humerus prosthesis without a cup					6	1
Complete anatomic prosthesis of humerus					1	1

The preoperative general condition of the majority of the patients was relatively good. Naturally enough, the Karnofsky scores and the VAS scores varied from patient to patient, depending on the site of the metastases (Table 3).

**Table 3:** Mean scores of pain intensity level rated by a VAS scale (mm) and the patients’ performance status level rated by a Karnofsky scale in relation to the location of the metastases (and the treatment applied)

	Proximal femur	Distal femur	Total femur	Proximal tibia	Proximal humerus	Total humerus
VAS scores	6.8	8.2	8.3	7.1	6.1	7.5
Karnofsky scores	53	40	40	45	65	60

**Fig. 3:**
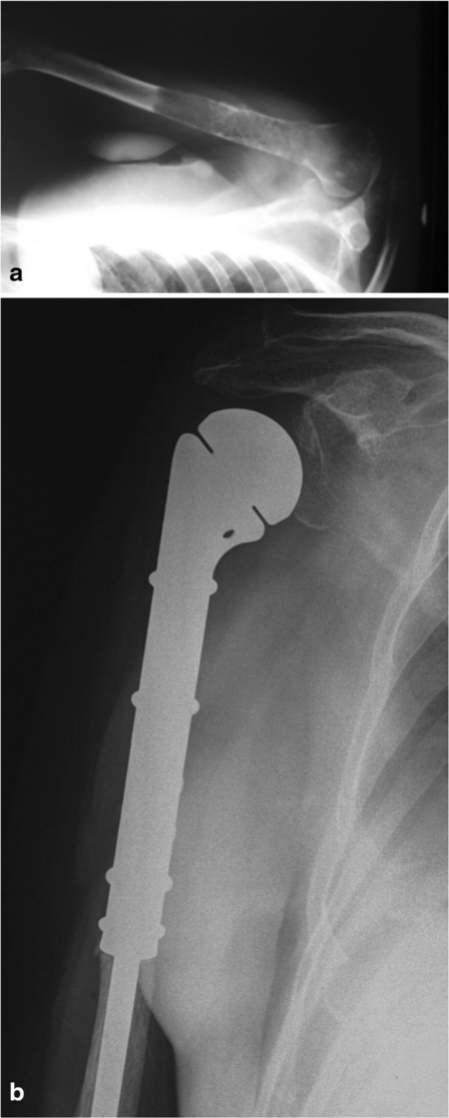
Radiographs of proximal humerus destruction by myeloma (**a**) and modular endoprosthetic replacement (**b**)

Patients with fractures of the lower extremity were not able to walk. The position of the limb was forced, and the presence of various deformities was detected (shortening, bent axis, thickened contour). Any attempts at moving resulted in a strong pain. No symptoms of ischemia or damage to peripheral nerves were detected. six patients with extensive lytic lesions in the lower limb were walking with the support of walking frames. The mobility of joints was reduced due to pain. Attempts made in a lying position at lifting the leg above the bed and extending the knee caused pain or were impossible. Metastases in the upper extremity did not affect the patients’ ability to walk. The patients were immobilized in Dessault vests or plaster splints. Also in this group, symptoms of ischemia or damage to peripheral nerves were not observed (Fig 3).

Bone and soft tissue resections were wide margin. There was no need to perform large vessel or nerve resections, with an exception of four patients with the axillary nerve situated in the tumor area. In any of the patients, the cancer did not infiltrate the skin, so it was not necessary to perform excision followed by reconstruction with flaps. The extent of bone resections ranging from the minimum of 6 cm in the humerus and the tibia and the maximum of 22 cm in the femur is shown below (Table 4).

**Table 4:** The extent of the bone resection versus the type of prosthesis used

	Proximal femur (mm)	Distal femur (mm)	Total femur	Proximal tibia (mm)	Proximal humerus (mm)	Total humerus
Minimum resection range	100	100	-	60	60	-
Maximum resection range	200	220	-	120	140	-
Mean range	160	140	-	80	100	-

In the postoperative period, a significant improvement in the quality of life was reported by all the patients as a result of reduced pain or its complete regression. The mean pain intensity score by VAS in patients after lower extremity surgeries was 3.8 and 3.1 in patients after upper extremity surgeries. The mean Karnofsky performance score was 65 in patients after lower extremity surgeries and 75 in patients after upper extremity surgeries.

In a group of 58 patients with metastases to lower extremities, 12 have been walking normally without the support of crutches. Those are the patients with implants in the following regions: the proximal area of the femur (6 patients), the distal area of the femur (5 patients), and the proximal area of the tibia (1 patient). Thirty-nine patients have been using only one crutch or stick when walking longer distances, while seven patients have been walking with both crutches.

In each patient, a significant decrease in the strength of muscles in the operated extremity was observed. The postoperative passive joint mobility was relatively good, but active joint mobility was significantly reduced in the patients who underwent the surgery of the arm. The mean flexion of the arm was 70°, the mean extension 47°, internal rotation achieved 30°, and external 15° of movement. Trendelenburg’s sign was clearly positive in patients after femur surgeries which was indicative of gluteal muscle dysfunction. The patients were able to manage the stairs either alternating feet (47 patients) or reverting to each step (11 patients). We observed no contractures of a knee joint which normally cause trouble getting up from a chair or make it impossible. The loss of range of knee motion was observed as extension deficit to 10° and flexion to 40°. During each of the proximal tibia resections, the patellar ligament was sutured to a reversed flap obtained from the medial head of the triceps muscle. The limb was immobilized in a brace for 6 weeks, and after that, time rehabilitation was started to improve the function of a knee extensor. Each time, a full knee extension range of motion was achieved.

**Fig. 4:**
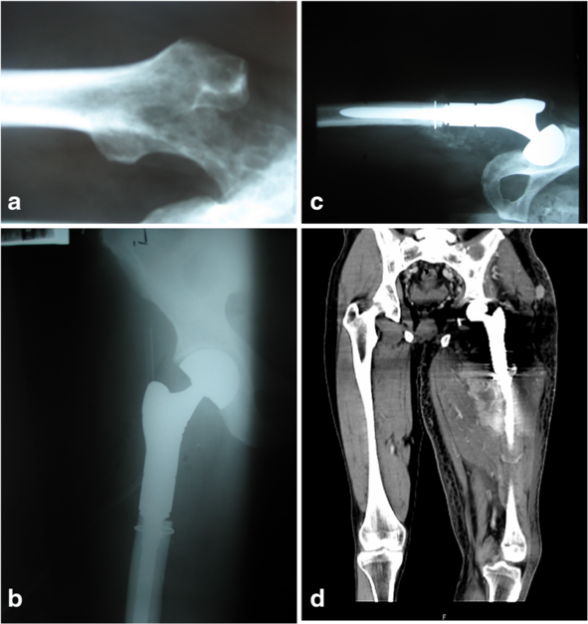
Chronologically arranged radiograms of kidney cancer metastases to the proximal femur (**a**) after resection of bone tumor and modular prosthesis implantation (**b**). Next, the radiogram (**c**) shows the postoperative recurrence of the disease. A coronal view from an MRI (**d**) shows a large tumor involving the whole thigh. There is visible damage to the femur and a loose prosthesis

No pathological fractures occurred in the same bone during the hospitalization. Complications involving loosening of an implant occurred in one patient (a case summarized below). Operative wound revision was necessary in two patients. The wounds were cleared from the granulation tissue, irrigated with BETADINE Solution, and the Garamycin sponge was implanted. Intraoperative evaluations were culture-negative. The wounds healed without further complications. Operative prosthesis revision was required in one case and simultaneous implantation of a new prosthesis was performed. The surgical wound healed by primary intention within 2 weeks after the revision procedure. In the remaining three cases, minor surgical wound infections were treated with intravenous antibiotic therapy without surgical intervention. No thromboembolic complications were observed. In one patient with renal clear-cell carcinoma, a massive local recurrence of the neoplasm occurred in the thigh 3 months after the surgery. The patient died after 5 months (Fig. 4).

In three other patients, minor local recurrences occurred. The patients were referred for palliative radiotherapy which resulted in the stability of the disease confirmed by the radiological examination. No dislocations of the prostheses were noted. One patient required LUMIC prosthesis implantation due to acetabular fracture after falling down.

## Discussion

Conventional osteosynthesis (fixation with an intramedullary nail or plates with or without bone cement) have represented a common option for surgical management of metastatic lesions [9–11]. Another possible choice of treatment that has been increasingly used is modular prostheses due to possibly long survival time in patients with myeloma and other metastatic cancers (breast, prostate, kidney, bowel, or thyroid cancer) [12–17].

**Fig. 5:**
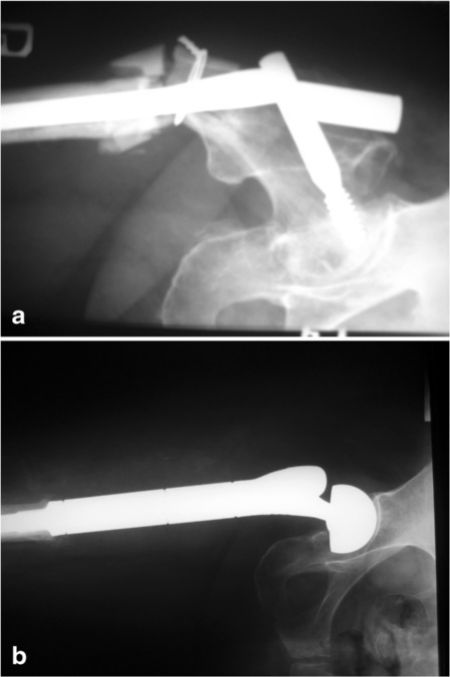
Failed fixation (gamma nail with PMMA) of a proximal femoral fracture due to breast cancer metastasis (**a**) and radiographs after modular endoprosthesis replacement (**b**)

With the progress in oncology, a significant number of malignant cancers, particularly those metastasizing to bones, have become chronic [18–21]. Technological developments in other fields, including material science, anesthesiology and surgical techniques, have made it possible to use large prostheses with an acceptable risk of complications. Materials used in a manufacturing process are increasingly better and biocompatible; therefore, the incidence of allergic reactions and intolerance symptoms has been lower. The comparisons of various surgical treatment options, osteosynthesis (with or without PMMA) and the prosthesis implantation, clearly indicate the superiority of the latter as presented in literature (Fig 5) [18, 20, 21].

The survival rate of the patients with modular prostheses after radical resections of metastatic tumors is higher when compared to the patients that underwent outdated standard treatment. The overall survival rate is sometimes as high as 37 months, which obviously varies in different cases, depending on the type of cancer, the grade of malignancy, the stage of the disease, and the methods of treatment [19–22].

Most studies have found that treatment results are better in cases where pathological fractures have never occurred which result from an easier technique of resection and a lower incidence of recurrences. Thus, it is recommended to perform a surgery in all the cases where metastases posing a risk of fracture were diagnosed. Clinical and radiological criteria for determining the risk of pathological fracture are now widely known. It should be kept in mind that a particularly high risk is associated with bones subject to heavy loading (the femur, the tibia, and the vertebral column) [18, 21, 23]. The recurrence rate in patients after long-bone metastatic tumor resections ranges from 4 to 28 %. The most important objective seems to be a wide margin with a cuff of healthy tissues which is a condition necessary for effective treatment [6, 8, 21, 22, 24].

One should consider the possibilities of adjuvant treatment, especially the use of radiation therapy and bisphosphonates. In the past, radiotherapy was a method of choice in treating bone metastases, particularly to the spine and the pelvis. Currently it is important as a method of reducing pain in 50–85 % of patients as well as reducing the incidence of local recurrences after surgeries. Bisphosphonates are also effective in pain reduction, and they significantly reduce the number of pathological fractures by 25–50 %. They are particularly often used in the treatment of metastatic breast cancer, prostate cancer, and multiple myeloma) [4, 6, 7, 25].

In the case of the metastases of kidney cancer, thyroid cancer, and myeloma, a trans-arterial embolization (TAE) can be performed. This procedure limits vascularization of the tumor, which results in a twofold or even threefold reduction in bleeding during the surgery, and the operation time is reduced by about 25 %. Many authors have confirmed the effectiveness of this treatment method in reducing pain and number of local recurrences after resection of the tumor. Reduced intraoperative bleeding allows for more precise preparation of tissues and resection of lesions. According to some authors, embolization increases the sensitivity of tumor cells to chemotherapy and radiation therapy. We have not performed preoperative embolization of tumors localized in the extremities at our department. This procedure has been performed exclusively when the tumor was localized in the pelvis and the spine [26–28].

The indications for amputation due to cancer metastases are extremely rare. It is performed in the case of large metastatic tumors infiltrating the vascular and nerve trunks or skin when limb-salvage treatment is not possible. An indication for amputation may be extensive inflammation of bone and soft tissues that is localized within the site of a former prosthetic implantation and which is impossible to treat [2, 3, 18].

**Fig. 6:**
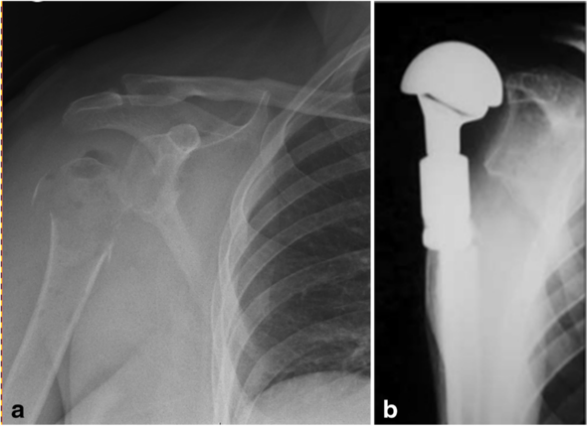
Radiographs of proximal humerus destruction by metastatic breast cancer (**a**) and after modular endoprosthetic replacement (**b**)

The present study clearly indicates that most metastases occur in the proximal area of the femur. Metastases in other sites are less frequent; very rarely do they occur in the area below the elbow or knee joint. Breast cancer is the most metastatic (Fig 6). Prostate cancer metastases, frequent as they are, rarely necessitate surgical treatment because of a relatively low risk of a fracture.

Most common complications that may follow modular alloplasty are surgical wound infections [29, 30]. Patients constitute a group at the highest risk of infectious and thromboembolic complications because the surgeries are most often urgent, therefore, MRSA screening or other pathogen detection tests are rarely carried out. There are no clear recommendations as to the routine local antibiotic therapy in the case of primary resection alloplasty. A rate of infectious complications ranges between 1.2 and 19.5 %. Preoperative radiotherapy is seen as one of the major risk factors for developing infections. Major risk factors include: decreased immunity as a result of neoplastic disease and chemotherapy, wide surgical approach with significant blood loss, and the size of metal implants. Worse still, the patients are usually elderly and with various general health problems.

Other potential complications include dislocation or loosening of the implants and periprosthetic fractures. Revision procedures are required in 3–17 % of patients [31, 32].

The procedures concerning preparation and implantation of GMRS and MUTARS prostheses differ. The MUTARS prostheses allow for a smooth rotation of the prosthesis stem. What is more, surgical management of the bone marrow canal is carried out not by reaming, but by rasping. The stems are bent, which minimizes the risk of damaging the cortical bone while driving them. The prostheses have a golden and silver coating, which reduces the incidence of infectious complications and allergic reactions.

Functional results of modular alloplasty are satisfactory, especially in patients after proximal and distal femur resections. Decreased gluteal muscles function was not a big problem to patients. Most of the patients walk efficiently without crutches. A slight limb length discrepancy was not noticed by the patients and did not affect their walking. The quality of life of the patients after metastasis resections and implantations of modular prostheses improved significantly. The VAS and Karnofsky scales showed clearly reduced pain and improved functioning.

## Conclusions

1. Modular tumor endoprosthesis may be an option in surgical treatment of long-bone metastases providing fast pain relief and early mobilization.
2. Radical metastatic tumor resection is a condition necessary for a good treatment outcome. It prevents local recurrences and damage to implant or its loosening.
3. Proximal humerus resections result in a reduced shoulder mobility and weakening of muscle strength which impair normal limb functions.
4. The incidence rate of infections in patients after modular prostheses implantations varies. Efforts should be directed at preventing infectious complications because they are very difficult to treat.

## Abbreviations

MRSA: methicillin-resistant *Staphylococcus aureus*
PMMA: polymethyl methacrylate
TAE: trans-arterial embolization
